# Supplementary material for: The influence of psychological capital on employment expectations of vocational undergraduate students: The chain mediating role of active coping style and educational flow experience
Source: PLoS One. 2025 Mar 17;20(3):e0319742. doi: 10.1371/journal.pone.0319742 (PMC11913298; doi:10.1371/journal.pone.0319742)
Supplement: S1 Research Questionnaire — (DOCX) [file pone.0319742.s001.docx]

| **Research Questionnaire** | | | | | | | | |
| --- | --- | --- | --- | --- | --- | --- | --- | --- |
| **Undergraduate Students' Psychological Capital, Employment Expectations, Educational Flow Experience, and Active coping style Questionnaire** | | | | | | | | |
| This survey questionnaire forms part of an academic inquiry aimed at examining the interrelation among Psychological Capital, Employment Expectations, Educational Flow Experience, and Active coping style of undergraduate students within higher education institutions in China.  Should you be an undergraduate at Guangdong Vocational and Technical University of Business and Technology, your participation in completing this questionnaire would be most beneficial. Be assured that this survey does not collect names or any other personally identifying information; hence, we entreat you to respond with honesty.  All information procured will be exclusively employed for the purposes of this scholarly research and will be subject to stringent confidentiality protocols. Your contribution is highly valued and instrumental to the success of this study.  Contact Information: Huang Zerui, 16635711417 Email: huangzrio@foxmail.com | | | | | | | | |
|  |  |  |  |  |  |  |  |  |
| Kindly appraise each statement and select the option that most closely aligns with your current circumstances. For each descriptor, assess its congruence with your situation and assign an appropriate score accordingly. There are no right or wrong responses in this evaluation. Should a descriptor be entirely incongruent with your reality, please assign a score of [1]; conversely, if a descriptor is in complete alignment with your situation, assign a score of [7].  For instance, should a statement describe your capacity to remain composed and seek solutions when confronted with challenges and this description resonates somewhat with your reality, you may opt for "Somewhat Agree" and allocate a score of [5]. | | | | | | | | |
| Psychological Capital | | Strongly disagree | Disagree | Somewhat disagree | Neither agree or disagree | Somewhat agree | Agree | Strongly agree |
|  |  | 1 | 2 | 3 | 4 | 5 | 6 | 7 |
| When facing difficulties, I calmly seek solutions. | | □ | □ | □ | □ | ✔ | □ | □ |
|  |  |  |  |  |  |  |  |  |
| **Section A: Psychological Capital** | | | | | | | | |
| Please select the option that best aligns with your actual circumstances based on your experiential reality. | | | | | | | | |
| Psychological Capital | | Strongly disagree | Disagree | Somewhat disagree | Neither agree or disagree | Somewhat agree | Agree | Strongly agree |
|  |  | 1 | 2 | 3 | 4 | 5 | 6 | 7 |
| A1 | Many people admire my talents. | □ | □ | □ | □ | □ | □ | □ |
| A2 | I don’t like to get angry. | □ | □ | □ | □ | □ | □ | □ |
| A3 | My insights and abilities exceed the average person. | □ | □ | □ | □ | □ | □ | □ |
| A4 | When faced with setbacks, I recover quickly. | □ | □ | □ | □ | □ | □ | □ |
| A5 | I am very confident in my abilities. | □ | □ | □ | □ | □ | □ | □ |
| A6 | I seldom care about the unhappiness in life. | □ | □ | □ | □ | □ | □ | □ |
| A7 | I always complete tasks outstandingly. | □ | □ | □ | □ | □ | □ | □ |
| A8 | Bad experiences depress me for a long time. | □ | □ | □ | □ | □ | □ | □ |
| A9 | When facing difficulties, I calmly seek solutions. | □ | □ | □ | □ | □ | □ | □ |
| A10 | I feel that I live a tiring life. | □ | □ | □ | □ | □ | □ | □ |
| A11 | I am willing to take on difficult and challenging work. | □ | □ | □ | □ | □ | □ | □ |
| A12 | When things don’t go my way, I tend to get downhearted. | □ | □ | □ | □ | □ | □ | □ |
| A13 | In adversity, I actively try different strategies. | □ | □ | □ | □ | □ | □ | □ |
| A14 | Under great pressure, I have trouble eating and sleeping well. | □ | □ | □ | □ | □ | □ | □ |
| A15 | I work or study actively to realize my dreams. | □ | □ | □ | □ | □ | □ | □ |
| A16 | When situations are uncertain, I always expect good outcomes. | □ | □ | □ | □ | □ | □ | □ |
| A17 | I am working hard to achieve my goals. | □ | □ | □ | □ | □ | □ | □ |
| A18 | I always see the positive side of things. | □ | □ | □ | □ | □ | □ | □ |
| A19 | I pursue my goals with confidence. | □ | □ | □ | □ | □ | □ | □ |
| A20 | I believe that there are still mostly good people in society. | □ | □ | □ | □ | □ | □ | □ |
| A21 | I have certain plans for my studies and life. | □ | □ | □ | □ | □ | □ | □ |
| A22 | Most of the time, I am spirited and lively. | □ | □ | □ | □ | □ | □ | □ |
| A23 | I am clear about the kind of life I want. | □ | □ | □ | □ | □ | □ | □ |
| A24 | I think life is beautiful. | □ | □ | □ | □ | □ | □ | □ |
| A25 | I don’t know what my life goals are. | □ | □ | □ | □ | □ | □ | □ |
| A26 | I feel that the future is full of hope. | □ | □ | □ | □ | □ | □ | □ |
|  |  |  |  |  |  |  |  |  |
| **Section B: Educational Flow Experience** | | | | | | | | |
| Please select the option that most accurately reflects your actual situation during participation in the learning activity. | | | | | | | | |
| Educational Flow Experience | | Strongly disagree | Disagree | Somewhat disagree | Neither agree or disagree | Somewhat agree | Agree | Strongly agree |
|  |  | 1 | 2 | 3 | 4 | 5 | 6 | 7 |
| B1 | I trust my ability to meet the high demands of the situation. | □ | □ | □ | □ | □ | □ | □ |
| B2 | I am wholly absorbed in what I am doing. | □ | □ | □ | □ | □ | □ | □ |
| B3 | I don’t care about what others may think of me. | □ | □ | □ | □ | □ | □ | □ |
| B4 | I have the feeling I am living a very exciting experience. | □ | □ | □ | □ | □ | □ | □ |
| B5 | I feel completely in control of my actions. | □ | □ | □ | □ | □ | □ | □ |
| B6 | I am losing track of time. | □ | □ | □ | □ | □ | □ | □ |
| B7 | I am not concerned about the judgement of others. | □ | □ | □ | □ | □ | □ | □ |
| B8 | This activity brings me a sense of well-being. | □ | □ | □ | □ | □ | □ | □ |
| B9 | At each step, I know exactly what I have to do. | □ | □ | □ | □ | □ | □ | □ |
| B10 | I am deeply focused on what I am doing. | □ | □ | □ | □ | □ | □ | □ |
| B11 | I am not worried about what others might think of me. | □ | □ | □ | □ | □ | □ | □ |
| B12 | When I talk about this activity, I feel such a deep emotion that I want to share it. | □ | □ | □ | □ | □ | □ | □ |
|  |  |  |  |  |  |  |  |  |
| **Section C: Employment Expectations** | | | | | | | | |
| Please select the option that best aligns with your actual circumstances based on your experiential reality. | | | | | | | | |
| Employment Expectations | | Strongly disagree | Disagree | Somewhat disagree | Neither agree or disagree | Somewhat agree | Agree | Strongly agree |
|  |  | 1 | 2 | 3 | 4 | 5 | 6 | 7 |
| How important is it to you to have a job which: | |  |  |  |  |  |  |  |
| C1 | requires originality and creativeness. | □ | □ | □ | □ | □ | □ | □ |
| C2 | makes use of your specific educational background. | □ | □ | □ | □ | □ | □ | □ |
| C3 | encourages continued development of knowledge and skills. | □ | □ | □ | □ | □ | □ | □ |
| C4 | is respected by other people. | □ | □ | □ | □ | □ | □ | □ |
| C5 | provides job security. | □ | □ | □ | □ | □ | □ | □ |
| C6 | provides the opportunity to earn a high income. | □ | □ | □ | □ | □ | □ | □ |
| C7 | makes a social contribution by the work you do. | □ | □ | □ | □ | □ | □ | □ |
| C8 | gives you the responsibility for taking risks. | □ | □ | □ | □ | □ | □ | □ |
| C9 | requires working on problems of central importance to the organization. | □ | □ | □ | □ | □ | □ | □ |
| C10 | involves working with congenial associates. | □ | □ | □ | □ | □ | □ | □ |
| C11 | provides ample leisure time off the job. | □ | □ | □ | □ | □ | □ | □ |
| C12 | provides change and variety in duties and activities. | □ | □ | □ | □ | □ | □ | □ |
| C13 | provides comfortable working conditions. | □ | □ | □ | □ | □ | □ | □ |
| C14 | permits advancement to high administrative responsibility. | □ | □ | □ | □ | □ | □ | □ |
| C15 | permits working independently. | □ | □ | □ | □ | □ | □ | □ |
| C16 | rewards good performance with recognition. | □ | □ | □ | □ | □ | □ | □ |
| C17 | requires supervising others. | □ | □ | □ | □ | □ | □ | □ |
| C18 | is intellectually stimulating. | □ | □ | □ | □ | □ | □ | □ |
| C19 | satisfies your cultural and aesthetic interests. | □ | □ | □ | □ | □ | □ | □ |
| C20 | has clear cut rules and procedures to follow. | □ | □ | □ | □ | □ | □ | □ |
| C21 | permits you to work for superiors you admire and respect. | □ | □ | □ | □ | □ | □ | □ |
| C22 | permits a regular routine in time and place of work. | □ | □ | □ | □ | □ | □ | □ |
| C23 | requires meeting and speaking with many other people. | □ | □ | □ | □ | □ | □ | □ |
| C24 | permits you to develop your own methods of doing the work. | □ | □ | □ | □ | □ | □ | □ |
| C25 | provides a feeling of accomplishment. | □ | □ | □ | □ | □ | □ | □ |
|  |  |  |  |  |  |  |  |  |
| **Section D: Active coping style** | | | | | | | | |
| Please select the option that best aligns with your actual circumstances based on your experiential reality. | | | | | | | | |
| Active coping style | | Strongly disagree | Disagree | Somewhat disagree | Neither agree or disagree | Somewhat agree | Agree | Strongly agree |
|  |  | 1 | 2 | 3 | 4 | 5 | 6 | 7 |
| D1 | I thought about what I would say or do. | □ | □ | □ | □ | □ | □ | □ |
| D2 | I talked to someone about my feelings. | □ | □ | □ | □ | □ | □ | □ |
| D3 | I made preparations to face the worst. | □ | □ | □ | □ | □ | □ | □ |
| D4 | I talked to people to understand more about the situation. | □ | □ | □ | □ | □ | □ | □ |
| D5 | I knew what needed to be done‚ so I worked extra hard to make things work. | □ | □ | □ | □ | □ | □ | □ |
| D6 | I expressed my feelings in one way or another. | □ | □ | □ | □ | □ | □ | □ |
| D7 | I made myself change or grow as an individual. | □ | □ | □ | □ | □ | □ | □ |
| D8 | I sought advice from relative or friends I respected. | □ | □ | □ | □ | □ | □ | □ |
|  |  |  |  |  |  |  |  |  |
| **Section E: Demographic** | | | | | | | | |
| E1 | Your Gender | □ | Male |  |  | □ | Female |  |
|  |  |  |  |  |  |  |  |  |
| E2 | Your Year in School | □ | Freshman |  |  | □ | Sophomore |  |
|  |  | □ | Junior |  |  | □ | Senior |  |
|  |  |  |  |  |  |  |  |  |
| E3 | Your Age | □ |  |  |  |  |  |  |
|  |  |  |  |  |  |  |  |  |
| E4 | Your Residential Area | □ | Urban |  |  | □ | Rural |  |
|  |  | □ | Suburban |  |  |  |  |  |
| Thank you for your cooperation! | | | | | | | | |
