# Supplementary material for: The influence of psychological capital on employment expectations of vocational undergraduate students: The chain mediating role of active coping style and educational flow experience
Source: PLoS One. 2025 Mar 17;20(3):e0319742. doi: 10.1371/journal.pone.0319742 (PMC11913298; doi:10.1371/journal.pone.0319742)
Supplement: S2 Appendix — (DOCX) [file pone.0319742.s002.docx]

**S2 Appendix**

| **Respondents' Descriptive Statistics** | | | | | |
| --- | --- | --- | --- | --- | --- |
| **Item** | **N** | **Min** | **Max** | **Mean** | **SD** |
| **Gender** | 693 | 1.00 | 2.00 | 1.456 | 0.498 |
| **Age** | 693 | 18.00 | 22.00 | 19.970 | 1.362 |
