# Supplementary material for: The influence of psychological capital on employment expectations of vocational undergraduate students: The chain mediating role of active coping style and educational flow experience
Source: PLoS One. 2025 Mar 17;20(3):e0319742. doi: 10.1371/journal.pone.0319742 (PMC11913298; doi:10.1371/journal.pone.0319742)
Supplement: S3 Appendix — (DOCX) [file pone.0319742.s003.docx]

**S3 Appendix**

| **Summary of Scale Items, Descriptive Statistics, and Reliability Coefficients** | | | | | |
| --- | --- | --- | --- | --- | --- |
| **Scale** | **Dimensions** | **Item Number** | **Mean** | **SD** | **Cronbach's Alpha** |
| **Psychological Capital** | Self-efficacy | A1, A2, A3, A4, A5, A6, A7 | 4.890 | 0.604 | 0.960 |
|  | Resilience | A8, A9, A10, A11, A12, A13, A14 | 5.340 | 0.512 | 0.953 |
|  | Optimism | A15, A16, A17, A18, A19, A20 | 5.353 | 0.545 | 0.948 |
|  | Hope | A21, A22, A23, A24, A25, A26 | 5.348 | 0.527 | 0.943 |
| **Educational Flow Experience** | Cognitive Control | B1, B5, B9 | 5.422 | 0.651 | 0.884 |
|  | Immersion and Time Transformation | B2, B6, B10 | 5.839 | 0.690 | 0.904 |
|  | Loss of Self-consciousness | B3, B7, B11 | 5.834 | 0.674 | 0.913 |
|  | Autotelic Experience | B4, B8, B12 | 5.832 | 0.672 | 0.893 |
| **Employment Expectations** | Job Attribute Preferences | C1, C3, C15, C18, C19, C21, C24, C25 | 4.363 | 0.716 | 0.941 |
|  | Career Values | C2, C5, C7, C16, C22, C23 | 5.168 | 0.691 | 0.853 |
|  | Long-term Career Success | C4, C6, C8, C9, C14, C17 | 4.943 | 0.667 | 0.924 |
|  | Comfortable Working Environment and Interpersonal Relationships | C10, C11, C12, C13, C20 | 5.124 | 0.661 | 0.942 |
| **Active Coping Style** | Rational Problem-solving | D1, D5, D9, D13 | 4.953 | 0.714 | 0.954 |
|  | Resigned Distancing | D2, D6, D10, D14 | 5.501 | 0.692 | 0.947 |
|  | Seeking Support and Ventilation | D3, D7, D11, D15 | 5.492 | 0.706 | 0.948 |
|  | Passive Wishful Thinking | D4, D8, D12, D16 | 5.435 | 0.686 | 0.953 |
