# Supplementary material for: The influence of psychological capital on employment expectations of vocational undergraduate students: The chain mediating role of active coping style and educational flow experience
Source: PLoS One. 2025 Mar 17;20(3):e0319742. doi: 10.1371/journal.pone.0319742 (PMC11913298; doi:10.1371/journal.pone.0319742)
Supplement: S4 Appendix — (DOCX) [file pone.0319742.s004.docx]

**S4 Appendix**

| **CFA Fit Indices by Scale** | | | | | |
| --- | --- | --- | --- | --- | --- |
| **Scale/ Subscale** | **χ^2^/df** | **RMSEA** | **CFI** | **NFI** | **GFI** |
| **Psychological Capital** | 2.728 | 0.049 | 0.979 | 0.967 | 0.917 |
| -Self-efficacy | 2.193 | 0.047 | 0.949 | 0.947 | 0.902 |
| -Resilience | 2.672 | 0.042 | 0.986 | 0.979 | 0.921 |
| -Optimism | 2.419 | 0.044 | 0.957 | 0.953 | 0.919 |
| -Hope | 2.713 | 0.043 | 0.966 | 0.964 | 0.917 |
| **Educational Flow Experience** | 2.775 | 0.048 | 0.989 | 0.983 | 0.965 |
| -Job Attribute Preferences | 2.732 | 0.045 | 0.988 | 0.982 | 0.961 |
| -Career Values | 2.424 | 0.042 | 0.990 | 0.984 | 0.965 |
| -Long-term Career Success | 2.653 | 0.047 | 0.987 | 0.980 | 0.963 |
| -Comfortable Working Environment and Interpersonal Relationships | 2.710 | 0.046 | 0.989 | 0.981 | 0.964 |
| **Employment Expectations** | 2.197 | 0.042 | 0.986 | 0.975 | 0.936 |
| -Cognitive Control | 2.151 | 0.041 | 0.987 | 0.975 | 0.940 |
| -Immersion and Time Transformation | 2.219 | 0.043 | 0.985 | 0.973 | 0.934 |
| -Loss of Self-consciousness | 2.172 | 0.042 | 0.986 | 0.974 | 0.937 |
| -Autotelic Experience | 2.248 | 0.044 | 0.984 | 0.972 | 0.931 |
| **Active Coping Style** | 2.751 | 0.049 | 0.986 | 0.979 | 0.952 |
| -Rational Problem-solving | 2.612 | 0.045 | 0.989 | 0.983 | 0.963 |
| -Resigned Distancing | 2.696 | 0.046 | 0.988 | 0.981 | 0.960 |
| -Seeking Support and Ventilation | 2.734 | 0.047 | 0.987 | 0.980 | 0.958 |
| -Passive Wishful Thinking | 2.722 | 0.046 | 0.988 | 0.981 | 0.959 |
| Notes: The Accepted Validity Values, χ^2^/df ≤ 3, RMSEA ≤ 0.05, CFI ≥ 0.90, NFI ≥ 0.90, GFI ≥ 0.90. | | | | | |
