# Supplementary material for: The influence of psychological capital on employment expectations of vocational undergraduate students: The chain mediating role of active coping style and educational flow experience
Source: PLoS One. 2025 Mar 17;20(3):e0319742. doi: 10.1371/journal.pone.0319742 (PMC11913298; doi:10.1371/journal.pone.0319742)
Supplement: Attachment — Submitted filename: 20250205Response to Reviewers.docx [file pone.0319742.s009.docx]

Response to editor

Dear Editor,

Thank you for your careful review and valuable feedback on our manuscript. We deeply appreciate your suggestions and have made comprehensive revisions to the paper accordingly. Below are our detailed responses to each of your comments:

**Comment 1:**

In the context of bootstrapping in SEM, the estimated values of the coefficients typically remain practically the same as the original estimates obtained from the model. However, what does change are the standard errors (SE) and confidence intervals (CI).

Therefore, I suggest that the authors remove Figures 2 and 3 and revise the manuscript's wording. Only as a theoretical model could Figure 1 remain. I believe that the information in Table 2 is sufficient to support the paths shown in Figure 1.

**Response:**

Thank you for your thoughtful suggestions regarding the use of bootstrapping in SEM and the feedback on the figures. We fully understand and agree with your point, particularly that while the estimated values of the coefficients typically remain unchanged in bootstrapping, the standard errors (SE) and confidence intervals (CI) do vary.

We concur with your suggestion that Figures 2 and 3 are not necessary in this context, as the information presented in Table 2 sufficiently supports the paths shown in Figure 1. In accordance with your recommendation, we have removed Figures 2 and 3 and made the necessary revisions to the manuscript, including the corresponding adjustments in the text (see revised manuscript, lines 312-326).

Additionally, the SEM-related terminology has been updated and modified throughout the manuscript, and the reference list has been adjusted for consistency (see references 69-88, lines 286, 300, 310, 314, 316, 351, 354, 356, 359, 368, 372, 377, 388, 390, 403, 406, 412).

Once again, we would like to express our sincere gratitude for your invaluable feedback, which has significantly improved the clarity and focus of our paper. We believe that these revisions have enhanced the overall quality of the manuscript, and we are grateful for your continued support throughout the process. Should you have any further questions or require additional modifications, please do not hesitate to contact us.

Sincerely,

Huang Zerui
