# Supplementary material for: The influence of psychological capital on employment expectations of vocational undergraduate students: The chain mediating role of active coping style and educational flow experience
Source: PLoS One. 2025 Mar 17;20(3):e0319742. doi: 10.1371/journal.pone.0319742 (PMC11913298; doi:10.1371/journal.pone.0319742)
Supplement: Attachment — Submitted filename: Response to Reviewers.docx [file pone.0319742.s006.docx]

Response to editor and reviewers

Dear editor and reviewers,

We are deeply grateful for your thorough review and valuable feedback on our manuscript. We have carefully considered all suggestions and have made comprehensive revisions to address your concerns and enhance the quality and academic rigor of the study. Below is a detailed response to your comments:

**Response to the editor**

1. Clarification of vocational studies

Comment:

The study should clarify whether vocational undergraduates are part of university studies, as this varies internationally. This distinction would prevent misunderstandings.

Response:

Thank you for this valuable suggestion. In the revised introduction, we have explicitly clarified that “Vocational undergraduate education is part of China’s higher education system, emphasizing practical skills development.” Additionally, we have incorporated authoritative references to strengthen this clarification (see revised manuscript, lines 49–51).

2. Sample representativeness

Comment:

The sample is limited to a single vocational university in Guangdong Province, which may not represent the broader population of vocational students in China. Future research should include a more diverse sample from different regions and types of vocational institutions.

Response:

We recognize the regional limitation of our sample. This has been further emphasized in the limitations section. We have also suggested that future research expand sample sources to include students from different regions and types of vocational institutions to validate the applicability of the findings (see revised manuscript, lines 372–375).

3. Measurement tools

Comment:

All scales used should be included in an appendix to increase transparency and facilitate replication. Evaluating the internal consistency of the scales using coefficients like Cronbach’s alpha (values above 0.7 indicate good reliability) is also recommended.

Response:

All measurement scales (psychological capital, employment expectations, educational flow experience, and active coping style) and their respective items have been included in the appendix (uploaded as supporting information files). Additionally, we have reported the internal consistency of each scale (Cronbach’s alpha > 0.7), confirming their reliability (see revised manuscript, lines 167–168, 177–178, 187–188, 197–198).

4. Construct validity

Comment:

Factor analysis should be used to confirm that the structure of the items aligns with the theoretical construct, ensuring the robustness of the study’s claims.

Response:

We appreciate this critical suggestion. In the methods section, we have added descriptions of Confirmatory Factor Analysis (CFA) and reported fit indices (e.g., CFI, RMSEA) for each variable. The results indicate that the factor structures align with theoretical constructs (see revised manuscript, lines 168–169, 178–179, 188–189, 198–199).

5. Methodology

Comment:

The methodology, including Structural Equation Modeling (SEM), needs a more detailed explanation. A comprehensive overview of the SEM process, assumptions, and steps taken to ensure accuracy would enhance understanding and trust in the research approach.

Response:

We have expanded the methodology section with a detailed explanation of the SEM process, including assumptions, hypotheses, and data handling steps. We have also elaborated on model fit indices to enhance the transparency and rigor of the research approach (see revised manuscript, lines 210–229).

6. Discussion

Comment:

The discussion should highlight the relevance of the results to educational policy, including recommendations for policy changes, practical applications, and implications for future research. The current results reflect only expectations rather than real behaviors. Connecting the results more closely with actual experiences would enhance the study’s practical value. Follow-up studies observing real behaviors in educational settings could provide more concrete insights.

Response:

In the discussion section, we have highlighted the policy implications of our findings. Specifically, we propose that university administrators prioritize the cultivation of psychological capital and suggest that practical activities (e.g., internships and project-based learning) be implemented to enhance students’ educational flow experiences. Furthermore, we emphasize the importance of observing real employment behaviors in future research to enrich the practical value of the study (see revised manuscript, lines 342–370).

We greatly appreciate your guidance, which has significantly improved the quality of our manuscript. If further revisions are needed, please do not hesitate to contact us.

**Response to reviewers**

**Reviewer #1**

Comment:

The study is rigorous, but the unique contribution to undergraduate career guidance could be better clarified.

Response:

Thank you for your positive feedback. We have expanded the "Implications" section to emphasize how our findings contribute to career guidance for vocational undergraduates. Specifically, we elaborated on the practical applications of cultivating psychological capital, enhancing educational flow experience, and supporting active coping styles. We also added actionable recommendations, such as implementing project-based learning and mentorship programs, to help practitioners effectively apply these strategies (see revised manuscript, lines 342–370).

**Reviewer #2**

Comment 1: The study’s contribution to existing knowledge is limited.

Response:

We respectfully disagree. Our study addresses an understudied population—vocational undergraduates—filling a gap in employment expectations literature. To further highlight this contribution, we have revised the discussion section to explicitly state how incorporating psychological capital, educational flow experience, and active coping style into a chain mediation model extends existing theoretical frameworks (see revised manuscript, lines 342–370).

Comment 2: The research design lacks comprehensiveness.

Response:

We appreciate your suggestion and have added more details to the methodology section. This includes an expanded explanation of SEM and Bootstrapping processes, model assumptions, fit indices, and analysis steps. We also provided additional validation of the scales’ reliability and validity, ensuring the research design’s rigor and clarity (see revised manuscript, lines 210–229).

Comment 3: The discussion does not adequately interpret findings or relate them to the research questions.

Response:

We have enriched the discussion section with more in-depth interpretations of our findings and their alignment with the research hypotheses. Additionally, we elaborated on the practical implications of the results for vocational undergraduate education practices, ensuring a stronger connection between the findings and the research questions (see revised manuscript, lines 342–370).

**Reviewer #3**

Comment 1: Can vocational education meet students' expectations in a competitive job market?

Response:

Thank you for this insightful question. In the discussion section, we added an analysis of the role of vocational education in addressing challenges in a tight job market. Specifically, we discussed how cultivating psychological capital and active coping strategies can help students enhance their competitiveness. We also emphasized the importance of aligning vocational education with industry demands and the necessity of further skill development to meet employment expectations (see revised manuscript, lines 342–370).

Comment 2: The definition of psychological capital in vocational education should be clarified.

Response:

We agree with this suggestion and have clarified the definition of psychological capital in the literature review. We differentiated its general meaning from its application in vocational education, providing a more precise context for our research (see revised manuscript, lines 95–100).

Comment 3: A discrepancy exists between the correlation and SEM results regarding the relationship between psychological capital and employment expectations.

Response:

Thank you for pointing this out. Correlation analysis reflects a simple linear relationship, whereas SEM path coefficients represent controlled causal relationships. Moreover, multicollinearity tests (correlation coefficients < 0.8; VIF < 5) confirmed no multicollinearity issues in the model. Thus, the SEM results are statistically robust and consistent with the correlation analysis in trend.

We hope our revisions and responses address all concerns. Thank you again for your valuable feedback, which has greatly enhanced the quality of our manuscript.

Sincerely,

Huang Zerui
