# Supplementary material for: The influence of psychological capital on employment expectations of vocational undergraduate students: The chain mediating role of active coping style and educational flow experience
Source: PLoS One. 2025 Mar 17;20(3):e0319742. doi: 10.1371/journal.pone.0319742 (PMC11913298; doi:10.1371/journal.pone.0319742)
Supplement: Attachment — Submitted filename: 250127Response to Reviewers.docx [file pone.0319742.s007.docx]

Response to editor and reviewers

Dear editor and reviewers,

Thank you for reviewing our manuscript and for the valuable feedback from you and the reviewers. We greatly appreciate these insightful suggestions and have made comprehensive revisions and additions to the manuscript. Below, we provide detailed responses to each point raised:

**Response to the editor**

**Comment 1:**

Since university students' job expectations are primarily shaped by the state of the economy and employment opportunities, it is important that the authors highlight early in the introduction the importance of also studying the influence of psychological capital on the formation of vocational undergraduate students' job expectations, which is the main objective of the paper. Some ideas from the discussion can be included, such as psychological capital, which includes aspects like resilience, self-esteem, hope, and optimism, all of which play a crucial role in how individuals perceive and might face their future job challenges.

**Response:**

Thank you for this insightful feedback and suggestion. We recognize the importance of emphasizing the role of psychological capital in shaping vocational undergraduates’ job expectations early in the introduction. To address this, we have revised the introduction to explicitly highlight the significance of psychological capital. Specifically, we incorporated ideas from the discussion, emphasizing how resilience, self-esteem, hope, and optimism influence individuals' perceptions of and responses to future job challenges. These adjustments underscore the core objective of our study and justify the importance of examining psychological capital alongside external factors such as economic conditions and employment opportunities (see revised manuscript, Lines 68-72, 84-90).

**Comment 2:**

In the revised version, there is still confusion because it is not clear what the difference is between a university degree, for example in computer science, and a vocational degree offered by a university. What degrees or programs are offered, for example? Could you provide the ISCED equivalent, as it is an internationally standardized measure? It is important to contextualize the paper in an international context since technical and vocational education is part of upper secondary education in most European countries.

**Response:**

Thank you for raising this critical point. We recognize that clarifying the distinctions between vocational and traditional university degrees is essential, particularly for an international audience. In the revised manuscript, we elaborated on the differences between vocational undergraduate education and traditional undergraduate education. For instance, vocational undergraduate education focuses on practical skill development and offers industry-aligned programs such as engineering technology, nursing, information technology, and logistics management, whereas traditional education emphasizes theoretical knowledge and research capabilities.

Additionally, we incorporated the ISCED classification to contextualize vocational undergraduate education internationally. Under ISCED, vocational undergraduate education corresponds to Level 6, which represents bachelor’s level technical and vocational education. This clarification enhances the paper's global relevance and helps international readers better understand the positioning of vocational undergraduate education in China (see revised manuscript, Lines 50-63).

**Comment 3:**

The literature review is still limited, as this section focuses more on the hypotheses. For example, expand on how psychological capital can influence entrepreneurial intentions. It could also be expanded to give more consistency to the hypotheses being tested. Hypothesis 1 should be highlighted more because, as indicated in the first point, a labor economist would say that employment expectations are formed based on the state of the economy and the employment opportunities offered to university graduates. The paper aims to demonstrate that psychological capital can also play a role here.

**Response:**

Thank you for your valuable feedback. We acknowledge the need to expand the literature review to provide more robust theoretical support for our hypotheses. In the revised manuscript, we have expanded this section by further exploring how psychological capital influences entrepreneurial intentions. Specifically, we included additional studies to explain how psychological capital enhances confidence, proactivity, and resilience, which in turn directly or indirectly influence entrepreneurial behavior.

We have also emphasized Hypothesis 1 more prominently, discussing in greater depth how psychological capital, as a positive internal psychological resource, significantly impacts the formation of employment expectations. This addition further highlights the unique contribution of our study in examining the role of psychological mechanisms in shaping job expectations, alongside external economic factors (see revised manuscript, Lines 123-134, 138-147, 150-174).

**Comment 4:**

Although the authors provide a Word document with the scale items, this is not sufficient. In the body of the manuscript, as an appendix after the bibliography, there should be a table with the items grouped by scales and subscales (dimensions) as they were finally used in the research. For all of them, descriptive statistics along with Cronbach's alpha coefficients should be provided.

**Response:**

Thank you for pointing this out. We understand the importance of providing a detailed presentation of the scales used in the study to enhance transparency and replicability. In the revised manuscript, we have added a table in the appendix section that organizes the scale items by dimensions, as used in the research. The table also includes descriptive statistics (e.g., means, standard deviations) and Cronbach’s alpha coefficients for each dimension, allowing readers to evaluate the reliability and quality of the scales used (see revised manuscript, Lines 224-225, Appendix B, Line 791).

**Comment 5:**

How was CFA executed? The authors state that “Confirmatory Factor Analysis (CFA) was performed to evaluate the convergent and discriminant validity of the measurement model, ensuring consistency between the theoretical constructs of the latent variables and the actual measurement data.” This is not proven. It should also appear in an appendix after the bibliography. CFA should confirm that the variables (dimensions) are indeed grouped into the factors they intend to measure. This is important to give credibility to the hypotheses being tested.

**Response:**

We appreciate your insightful comments on the CFA section. We recognize that providing detailed information about the CFA process and results is crucial for demonstrating the validity of the measurement model. In the revised manuscript, we elaborated on how CFA was conducted. Specifically, we performed CFA separately for each scale (Psychological Capital, Employment Expectations, Educational Flow Experience, and Active Coping Style) to verify convergent and discriminant validity. These results ensure that the items within each scale appropriately measure their intended dimensions, and we included the CFA results, factor loadings, and fit indices in Appendix C (see revised manuscript, Lines 226-231, Appendix C, Line 792).

Thank you again for your valuable feedback, which has greatly improved the quality and rigor of our paper. Please feel free to reach out if there are any further questions or additional revisions required.

**Response to reviewers**

**Reviewer #2**

**Comment:**

In order to provide support for the findings of the research, it is necessary to conduct an in-depth analysis and discussion of the demographic profile of the respondents. Therefore, it is suggested that the author(s) include a table that provides a summary of the respondents' profiles.

**Response:**

Thank you for your constructive suggestion. We agree that analyzing and discussing the demographic profile of respondents is essential for supporting the research findings. In the revised manuscript, we included a table summarizing the demographic characteristics of the respondents, such as gender and age, along with descriptive statistics. This addition aims to help readers better understand the research context and sample representativeness (see revised manuscript, Lines 192-198, Table 1).

**Reviewer #3**

**Comment:**

The authors have addressed the issues raised by the reviewer, however, a more elaborate analysis of the subject is overdue, and could partaken in later studies. This research identifies the key factors that influence the job expectations of vocational undergraduate students in China. It also elucidates the role of psychological capital in employment prospects of vocational students. More the psychological capital, better it is for the vocational students to obtain employments, as this theory has been extended, proposed, with hypotheses designed to prove the theoretical claim. Rigorous statistical analyses have been performed, and the data sources has been provided by the author.

**Response:**

Thank you for your positive evaluation and thoughtful suggestions. We are pleased to know that you found the topic and statistical analyses well-presented and the role of psychological capital effectively elucidated.

We fully agree that further exploration and elaboration are warranted in future studies. Potential future research directions include incorporating more diverse samples, such as vocational undergraduates from various regions and academic disciplines, to enhance the generalizability of the findings. Additionally, longitudinal research could explore the dynamic nature of psychological capital and its long-term effects on employment expectations. Qualitative methods could also provide deeper insights into the psychological mechanisms and practical experiences of vocational undergraduates in their job search processes.

We are confident that these future directions will contribute to a more comprehensive understanding of the factors influencing employment expectations among vocational undergraduates. Thank you again for your recognition and valuable suggestions.

We have carefully addressed each reviewer’s comments and made corresponding revisions to improve the manuscript's scientific rigor, completeness, and academic value. Thank you again for your time and constructive feedback.

Sincerely,

Huang Zerui
