# Supplementary material for: The influence of psychological capital on employment expectations of vocational undergraduate students: The chain mediating role of active coping style and educational flow experience
Source: PLoS One. 2025 Mar 17;20(3):e0319742. doi: 10.1371/journal.pone.0319742 (PMC11913298; doi:10.1371/journal.pone.0319742)
Supplement: Attachment — Submitted filename: 20250131Response to Reviewers.docx [file pone.0319742.s008.docx]

Response to editor

Dear Editor,

Thank you for your thoughtful review and feedback on our manuscript. We greatly appreciate the time and effort you have dedicated to reviewing our work, and we have carefully considered all of your suggestions. We have made comprehensive revisions and additions to the manuscript, and below are our detailed responses to each point raised:

**Response to editor**

**Comment 1:**

In Appendix C, CFA Fit Indices should also be included for the subscales. Additionally, the accepted validity values for these indices should be indicated in a table footnote.

**Response:**

We sincerely thank you for your meticulous review and valuable suggestion. In response to your comment, we have included the CFA fit indices for all subscales in Appendix C. Additionally, we have clearly indicated the accepted validity values for these indices in the table footnote (please see line 782, Appendix C for details).

**Comment 2:**

On line 330, where does the 0.85 come from?

**Response:**

We deeply appreciate your careful attention to the details of our manuscript. Regarding the 0.85 value mentioned in line 330, we acknowledge that this was an error during the data entry process. Upon review, we have confirmed that the correct value should be 0.587, which is consistent with the path coefficient shown in Figure 3. We have made the necessary corrections in the revised manuscript to ensure consistency and accuracy of the values (please see line 330 of the revised version). We apologize for any confusion this may have caused and sincerely thank you for bringing this to our attention.

**Comment 3:**

According to lines 340-341: “The direct effect of psychological capital on employment expectations was significant (γ = 0.471, p < 0.001).” However, in Fig. 3 it says 0.587***.

**Response:**

We would like to express our heartfelt thanks for your careful examination of our paper. The path coefficient of 0.471 mentioned in lines 340-341 corresponds to the results based on the Bootstrapping method used to report the indirect effects. This coefficient reflects the average value obtained through 5000 resamples and emphasizes the stability and significance of the path coefficient. On the other hand, the path coefficient of 0.587 shown in Fig. 3 is derived from the SEM model under optimal estimation conditions, which represents the best fit of the model.

The difference between these two path coefficients does not represent a conflict; rather, it is due to the different calculation processes of the two methods. SEM uses optimal fit estimation and focuses on obtaining the best path coefficients from the overall data. Bootstrapping, by contrast, calculates the average value of the path coefficient through multiple resampling, placing greater emphasis on the variability and confidence intervals of the coefficient, which is why the values may differ.

Given this distinction, we believe it is more appropriate to maintain the value (γ = 0.471, p < 0.001) from Table 2 in this section, as it is derived from the Bootstrapping method and reflects the indirect effects. All discussions regarding the indirect effects in the manuscript are based on results from the Bootstrapping method.

**Comment 4:**

The indirect effect PC→EFE→EE in Fig. 3, is it obtained by multiplying 0.531 by 0.343? Then the result is 0.182. But in Table 2 it says 0.124.

**Response:**

We greatly appreciate your careful review and inquiry regarding the indirect effect in our study. Concerning the indirect effect of PC→EFE→EE, if we were to simply multiply the two path coefficients of 0.531 and 0.343, the resulting value would indeed be 0.182. However, the value reported in Table 2 is 0.124, and this discrepancy arises from the use of two different calculation methods. As discussed in response to Comment 3, the difference stems from the Bootstrapping method and the traditional SEM model estimation approach.

The indirect effect of 0.124 reported through Bootstrapping is derived from the average path coefficient calculated over 5000 resamples and takes into account the variability and uncertainty of the path coefficients. Thus, Bootstrapping better reflects the instability in the data and the actual distribution of the path coefficients, whereas traditional SEM path coefficient calculations do not consider these factors, resulting in a slightly higher value.

For the indirect effect section of the manuscript, we have used the Bootstrapping method, and we believe it is appropriate to maintain the value of 0.124 from Table 2 since it reflects the result obtained through this method. Additionally, the use of both SEM and Bootstrapping methods in our study serves to further validate the relationships between the variables, and the results from both methods converge, demonstrating that the path relationships in the model are stable and significant.

**Comment 5:**

I think that "Job Attribute Preferences" seems to be the most appropriate term to summarize items C1-C25, as it directly focuses on the job characteristics that students value. It's just a suggestion.

**Response:**

We sincerely appreciate your thoughtful suggestion regarding the terminology. After careful discussion and further consideration, we agree that the term "Job Attribute Preferences" is indeed highly appropriate as it accurately encapsulates the core content of items C1-C25, especially since these items directly reflect students' value of job characteristics. Consequently, we have revised the manuscript to ensure consistency and clarity in the use of this term, making the necessary adjustments throughout the text (please see lines 244-245 of the revised manuscript, as well as lines 781-782, Appendices B and C).

We would like to express our sincere gratitude once again to you and the reviewers for your invaluable feedback on our manuscript. Your suggestions have significantly enhanced the quality and rigor of our research. Should there be any further questions or areas requiring additional revisions, please do not hesitate to contact us.

Yours sincerely,

Huang Zerui
